# Domestic Cat Hepadnavirus and Pathogenic Retroviruses; A Sero-Molecular Survey of Cats in Santiago, Chile

**DOI:** 10.3390/v16010046

**Published:** 2023-12-27

**Authors:** Yan Ru Choi, María Paz Iturriaga, Omid Nekouei, Thomas Tu, Kate Van Brussel, Vanessa R. Barrs, Julia A. Beatty

**Affiliations:** 1 Department of Veterinary Clinical Sciences, Jockey Club College of Veterinary Medicine and Life Sciences, City University of Hong Kong, Hong Kong; yrchoi@cityu.edu.hk; 2 Centre for Animal Health and Welfare, City University of Hong Kong, Hong Kong; 3 Escuela de Medicina Veterinaria, Facultad de Ciencias de la Vida, Universidad Andrés Bello, Santiago 7550196, Chile; maria.iturriaga@unab.cl; 4 Department of Infectious Diseases and Public Health, Jockey Club College of Veterinary Medicine and Life, Hong Kong; omid.nekouei@cityu.edu.hk; 5 Storr Liver Centre, Westmead Clinical School and Westmead Institute for Medical Research, Faculty of Medicine and Health, The University of Sydney, Westmead, NSW 2145, Australia; t.tu@sydney.edu.au; 6 Sydney Institute for Infectious Diseases, University of Sydney at Westmead Hospital, Westmead, NSW 2145, Australia; 7 Department of Biology, University of Massachusetts, Boston, MA 02125, USA; kate.vanbrussel@umb.edu

**Keywords:** cat, hepadnavirus, feline leukaemia virus, feline immunodeficiency virus, South America, progressive

## Abstract

Cat ownership is common in Chile, but data on the regional prevalence of infectious agents are limited. A sero-molecular survey of 120 client- or shelter-owned domestic cats in greater Santiago was performed. Whole blood DNA was tested for the novel hepatitis-B-like virus, domestic cat hepadnavirus (DCH) by conventional PCR (cPCR) and quantitative PCR (qPCR), and for feline leukaemia virus (FeLV) by qPCR. Point-of-care serology for FeLV p27 antigen and antibodies recognising feline immunodeficiency virus (FIV) p15 and p24 was performed. DCH DNA was detected in the serum of 2/120 cats (1.67%). Sequencing and phylogenetic analysis showed that the DCH detected in Chile occupies a position outside the main clustering of DCH in the near-complete genome tree. Progressive (antigen-positive, provirus-positive) and regressive (antigen-negative, provirus-positive) FeLV infections were identified in 6/120 (5%) and 9/120 (7.5%) of cats. A total of 2/120 (1.7%) cats had dual FeLV/FIV infection, and another 2 cats had FIV infection alone. This study shows that the global footprint of DCH includes South America with a low molecular frequency in Chile, similar to that reported in the USA. Progressive FeLV infection is relatively common in urban Chile, and male cats are at greater risk than females. Testing and control measures for pathogenic retroviruses are indicated. The potential impact of FeLV, FIV and DCH on Chile’s wildcat species is worthy of further investigation.

## 1. Introduction

A global view of companion animal virus epidemiology informs public health, wildlife conservation and the veterinary industry. Chile is home to an estimated 12.5 million owned and 4 million unowned dogs and cats [1], and a population of over 17 million people, 40% of whom live in the Greater Santiago area [2,3]. Information for stakeholders on the presence and prevalence of feline viruses circulating in Chile is currently sparse.

Domestic cat hepadnavirus (DCH), an Orthohepadnavirus related to hepatitis-B virus of humans, was discovered in Australia in 2018 [4]. DCH-infected cats have since been detected in many regions including Italy [5], Thailand [6], Malaysia, Hong Kong, Japan, the USA, the UK, and Turkey [5,6,7,8,9,10,11]. The frequency of detection of DCH DNA in blood varies from less than 1% to 12.4% depending on the cat population studied [6,10]. Hepatitis-B virus and other viruses in the genus *Orthohepadnaviridae* can cause serious liver disease in their hosts. Whether DCH behaves similarly in cats is under investigation, although a role for the virus in some cases of feline hepatocellular carcinoma and chronic hepatitis seems likely. To our knowledge, no DCH infections of cats from South America have been reported.

The retroviruses feline leukemia virus (FeLV) and feline immunodeficiency virus (FIV) are common pathogens of domestic cats. Progressive infection with the gammaretrovirus FeLV infection, diagnosed by persistent p27 antigenemia and provirus detection), carries a poor prognosis with most cats succumbing to FeLV-associated diseases in month to years [12,13]. Since the discovery of FeLV almost 60 years ago, changes in cat management, the identification of persistently infected cats (which shed virus), and the availability of effective FeLV vaccines have seen the prevalence of infection decline in many areas [14]. However, in other regions, including southern Italy and Brazil, progressive FeLV infection and associated disease remains common [15,16,17].

The lentivirus FIV infects cats around the world, with increasing age, male sex, entire/intact status, poor health and access to outdoors being consistent risk factors [18]. FIV infection causes immune dysfunction in cats, but clinical immunosuppression seems to be the exception rather than the rule. Survival analyses suggest that FIV-associated mortality is uncommon [19], but morbidity from FIV infection is more difficult to evaluate. Coinfection with both FIV and FeLV carries a much worse prognosis than either infection alone [19]. Detection of anti-FIV antibodies is a reliable marker of FIV infection in most cases and qPCR is available when confirmation is required. The limited quantity of published data regarding FeLV and FIV infections of domestic cats in Chile is a barrier to informed recommendations on testing and control of these retroviruses in the region.

The aim of this study was to determine the presence, prevalence, and risk factors for DCH, progressive and regressive FeLV infection, and FIV infection in cats from the Metropolitan area of Santiago de Chile.

## 2. Materials and Methods

### 2.1. Ethics Statement

This study was approved by the Bioethics Committee of the Faculty of Life Sciences in accordance with the University Decree 1939/2012, 2084/2014 and resolution 89584/2018 of the University Andrés Bello, September 2018.

### 2.2. Population Sampled and Data Collection

Paired serum and whole blood samples were obtained with owner consent from cats visiting 3 clinics and 1 shelter in the Metropolitan area of Santiago de Chile and stored at −80 °C. The sample collection period was 1 November 2021 to 30 April 2022. Age, sex, neuter status, breed, outdoor access (yes or no) and ownership (client or shelter) were recorded for each sample. In addition, health status was categorised as healthy or sick by a veterinarian on the basis of clinical information available to them at the time of sampling.

### 2.3. Serology

Sera were tested for FeLV p27 antigen and antibodies recognising FIV p15 and p24 using a commercial point-of care (POC) test (SNAP FIV/FeLV combo; IDEXX, Westbrook, ME, USA) according to the manufacturer’s instructions.

### 2.4. Molecular Testing

DNA was extracted from 200 µL of whole blood for FeLV provirus and DCH testing using the QIAamp^®^ MinElute^®^ Virus kit (Qiagen GmbH, Hilden, Germany) as per the manufacturer’s instructions, using an elution volume of 50 μL. The presence of amplifiable DNA was confirmed using conventional PCR (cPCR) for glyceraldehyde 3-phosphate dehydrogenase (GAPDH). All samples tested positive for GAPDH.

#### 2.4.1. Domestic Cat Hepadnavirus Quantitative and Conventional PCR Testing

DCH-specific qPCR and cPCR were performed on all samples, as described previously [4,5]. For qPCR, briefly, 25 μL reactions were prepared comprising 100 ng template DNA or 10 μL of plasmid standard (containing a 1.4 kb fragment of the polymerase region of the Australian reference strain AUS/2016/Sydney) [5], and 12.5 μL of master mix (IQ Supermix; Bio-Rad Pacific Limited, Hong Kong), containing 600 nM of each primer (Table 1) and 200 nM of probe. Reactions were run using a Bio-Rad CFX96 real-time PCR machine. A sample was defined as positive if ≥10 copies of DCH DNA were detected in ≥2 (of 3) replicates with a Ct value of ≤38.5. The cut-off was 0.980 for R-squared, and 90–110% for efficiency.

DCH-specific cPCR reactions contained 2 uL of template DNA, DreamTaq™ Hot Start Green DNA Polymerase (Thermo Fisher Scientific, Graciuno, Vilnius, Lithuania), dNTP (Thermo Fisher Scientific, Graciuno, Vilnius, Lithuania) at a final concentration of 200 μM and a final primer concentration of 300 nM (Table 1). No-template (molecular-grade water) and positive controls (DCH-positive whole-blood-derived DNA) were included in all cPCR assays. Products were resolved using 1.5% agarose gel electrophoresis. Sanger sequencing was performed (BGI Genomics, Hong Kong) and Geneious software (version 2023.1.1) used to generate the consensus sequence. The sequences obtained on PCR from were compared with known sequences by Basic Local Alignment Search Tool (BLAST+ 2.14.0) analysis against the NCBI database [20].

#### 2.4.2. DCH Genome Sequencing and Analysis

For samples testing DCH-positive by cPCR and/or qPCR, genome sequencing was carried out using overlapping nested PCRs (nPCR) designed here, and a panhepadnavirus nPCR described previously [21,22]. Primers and PCR conditions for the PCRs are shown in Table 2. Each reaction contained 1 μL of template DNA, DreamTaq™ Hot Start Green DNA Polymerase (Thermo Fisher Scientific, Graciuno, Vilnius, Lithuania), dNTP (Thermo Fisher Scientific, Graciuno, Vilnius, Lithuania) at a final concentration of 200 μM, and a final primer concentration of 300 nM. One μL of the PCR product from the first round was used as template for the second round. No-template (molecular-grade water) and positive controls (DCH-positive whole-blood-derived DNA) were included in all cPCR assays. Products were resolved using 1.5% agarose gel electrophoresis. Sanger sequencing was performed (BGI Genomics, Hong Kong) and Geneious software (version 2023.1.1) used to assemble the sequences and annotate ORFs using publicly available domestic cat hepadnavirus sequences.

Nucleotide sequences for all domestic cat hepadnaviruses were downloaded from NCBI GenBank and CD-HIT version 4.8.1 [23] was used to remove identical sequences, using a threshold of 1.0. The L–INS–I algorithm in MAFFT version 7.481 was used for nucelotide alignments of the near-complete genome, polymerase, surface and core proteins [24]. The best-fit models of nucleotide substitution were determined using the model finder program in IQ-TREE version 2.1.3 [25,26]. Maximum-likelihood trees using the nearest neighbour interchange and ultrafast bootstrapping with 1000 replicates to indicate nodal support were also created in IQ-TREE v 2.1.3 [27].

**Table 1:** Published PCR primers and conditions used in this study for PCR assays.

Assay	Target Gene (Product Size)	Name	Purpose	Sequence	Cycling Conditions
DCH qPCR (Lanave et al. 2019) [5]	Polymerase (132 bp)	FHBV-for	Forward	CGTCATCATGGGTTTAGGAA	Initial Activation: 95 °C, 3 min Denaturation: 95 °C, 10 s Annealing-extension: 60 °C for 30 s40 cycles
		FHBV-rev	Reverse	TCCATATAAGCAAACACCATACAAT	
		FHBV-prob	Probe	[FAM]TCCTCCTAACCATTGAAGCCAGACTACT [QSY]	
DCH cPCR (Aghazadeh et al. 2018) [4]	Core (258 bp)	Hgap-F	Forward	CTAGAATGGCTACATGGGTTAG	Initial denaturation: 95 °C, 1 min Denaturation: 95 °C, 30 s Annealing: 55 °C, 30 s Extension: 72 °C, 1 min, 35 cycles Final extension: 72 °C, 5 min
		Hgap-R	Reverse	GTGCTCTGATAACCGTATGCTC	
FeLV nested PCR (Miyazawa et al. 1997) [28]	U3-gag region (1st round: 770 bp 2nd round: 601 bp)	U3-F(1)	1st round Forward	ACAGCAGAAGTTTCAAGGCC	Initial denaturation: 95 °C, 2 min Denaturation: 95 °C, 45 s Annealing: 55 °C, 30 s Extension: 72 °C, 1 min, 35 cycles Final extension: 72 °C, 10 min
		G-R(1)	1st round Reverse	GACCAGTGATCAAGGGTGAG	
		U3-F(2)	2nd round Forward	GCTCCCCAGTTGACCAGAGT	
		G-R(2)	2nd round Reverse	GCTTCGGTACCAAACCGAAA	

**Table 2:** Nested PCR primers and conditions for sequencing DCH genome from a cat in the Metropolitan area of Santiago de Chile.

Primer Set	Nested PCR Round	Name	Primer Binding Site	Sequence	Product Size (bp)	Cycling Conditions		
			(Numbering Based on NC040719)			Denaturation	Annealing	Extension
1	1st round	2239OF_1	2234-2253	CAGAAGACGTACTCCCTYKC	599	95 °C, 30 s	57 °C, 30 s	72 °C, 1 min
		2842OR_1	2832-2810	TCYTTGAGGGGATTGTGRTCGAA				
	2nd round	2264IF_1	2259-2278	AGACGCAGATCTCAATCTYC	456	95 °C, 30 s	55 °C, 30 s	72 °C, 1 min
		2724IR_1	2714-2694	AARTGCAGTTTTTGTTCCCAG				
2	1st round	2677OF_2	2676-2698	TAATGGACAATTATACCCCTGGG	575	95 °C, 30 s	55 °C, 30 s	72 °C, 1 min
		63OR_2	63-43	CMAGAAAGATCCCACCTGTAA				
	2nd round	2695IF_2	2694-2714	CTGGGAACAAAAACTGCAYTT	444	95 °C, 30 s	58 °C, 30 s	72 °C, 1 min
		3138IR_2	3137-3118	CAGTAGGGGCCACAGTAGTC				
3	1st round	2914OF_3	2913-2932	CTCTCAGTTTCGCAACCCAG	609	95 °C, 30 s	55 °C, 30 s	72 °C, 1 min
		334OR_3	2996-3015	CAGGAGGAAAAGGACATACC				
	2nd round	2997IF_3	262-240	CCTTTAACTCCTCCTGTTCG	454	95 °C, 30 s	58 °C, 30 s	72 °C, 1 min
		262IR_3	334-315	CCAGGAGCAAGAGGTAAATGATA				
4	1st round	FHBV-F	458-478	CGTCATCATGGGCTTTAGGAA	575	95 °C, 30 s	57 °C, 30 s	72 °C, 1 min
		1032OR_4	1032-1012	CAAACACCTGGCARGACATTG				
	2nd round	572IF_4	572-594	GGTGTTTGCTTATATGGATGAYG	428	95 °C, 30 s	57 °C, 30 s	72 °C, 1 min
		999IR_4	999-979	GRACAAAGTACAAGTGTGTGT				
5	1st round	687F_2^+^	687-706	ACAAAAACCAAACGCTGGGG	811	95 °C, 30 s	55 °C, 30 s	72 °C, 1 min
		1497OR_5	1445-1428	GGCACRAGTCCAATCATG				
	2nd round	895OF_5	895-915	CTTTRATGCCTTTGTATACCG	551	95 °C, 30 s	57 °C, 30 s	72 °C, 1 min
		1445IR_5	1497-1480	TGAARCGAAGGACACACG				
6	1st round	1752OF_7	1751-1770	GGACATTGACCCTTATAAAG	600	95 °C, 30 s	53 °C, 30 s	72 °C, 1 min
		2351OR_7	2350-2332	TCAAAGACCCCCATTTATG				
	2nd round	1798IF_7	1797-1816	TCTTTTTTGCCGTCTGACTT	503	95 °C, 30 s	56 °C, 30 s	72 °C, 1 min
		2300IR_7	2299-2282	GATTGAGARCGTCTGCGA				

F_ denotes forward and R_ demotes reverse primers.

#### 2.4.3. FeLV Quantitative and Nested PCR Testing

Whole blood DNA samples were tested by qPCR targeting the U3 region of FeLV at a commercial laboratory (Asia Vet Diagnotics Ltd., Hong Kong, SAR, China). In addition, a subset of qPCR positive samples was tested using nPCR for FeLV as previously described [28]. In brief, each reaction contained 1 μL of template, DreamTaq™ Hot Start Green DNA Polymerase (Thermo Fisher Scientific, Graciuno, Vilnius, Lithuania), dNTP (Thermo Fisher Scientific, Graciuno, Vilnius, Lithuania) at a final concentration of 200 μM and a final primer concentration of 300 nM. In the nPCR, 2 μL of the PCR product from the first round was used as template for the second round. Primers and cycling conditions are shown in Table 1. No-template (molecular-grade water) and positive controls (FeLV-positive whole-blood-derived DNA) were included in all cPCR assays. Products were resolved using 1.5% agarose gel electrophoresis and sequencing analysis of PCR products was performed as described in Section 2.4.1.

#### 2.4.4. Interpretation of Infection Status

A positive result for DCH DNA was considered to indicate DCH infection. Cats testing FeLV antigen-negative and provirus-positive were defined as regressively infected. Cats testing FeLV antigen-positive and provirus-positive were assumed to have progressive FeLV infection, although longitudinal testing to confirm the outcome was not available. FeLV antigen-positive and provirus-negative was defined as a false-positive antigen test result, although rare, atypical focal infection could not be completely ruled out. FIV seropositivity was considered to be a marker for FIV infection since none of the client-owned cats in this study had been vaccinated against FIV and FIV vaccination is not available in Chile.

### 2.5. Statistical Analyses

All data management and analyses were performed using Stata v17 (Stata Corp LLC, College Station, TX, USA). Descriptive statistics were generated for all variables. The Ct-value distributions were compared between progressive and regressive FeLV infections using the Mann–Whitney U test. The associations between the independent variables of interest (source, sex, age, outdoor access, health status) and FeLV progressive infection were evaluated using Fisher’s exact test, with the significance level set at 0.05.

## 3. Results

### 3.1. Population Characteristics

In total, 120 individuals were available to study. The population comprised mixed-breed cats (119/120, 99.2%) except for a single purebred cat (Scottish Fold), with similar numbers of females (*n* = 66) and males (*n* = 56; female:male ratio: 1.18:1), and median age of 6 years old (range 1–21 years). Five cats were intact/entire (four female, one male) and the remainder had been neutered. There were 95 privately owned and 25 shelter-owned cats.

### 3.2. Domestic Cat Hepadnavirus Detection and Sequencing in Chilean Cats

DCH was detected in 1 of 120 samples by qPCR, with a virus load of 1.46 × 10^3^ copies/mL of blood, and in 2 of 120 (1.67%) samples by cPCR, 1 of which was the qPCR positive sample. Both DCH positive cats were estimated to be over 7 years of age and were housed in the same shelter where they shared a common area. The qPCR/cPCR DCH positive cat was assessed as “healthy”; the cat testing DCH positive on cPCR alone had chronic stomatitis and serum biochemistry was normal aside from hyperglobulinaemia.

From the sample testing positive on both DCH cPCR and qPCR, 2817 bp of the full virus sequence (approximately 88%) was obtained using overlapping nPCR. Despite multiple attempts, the full genome, including most of the X-protein, could not be sequenced. The partial DCH sequence was deposited in Genbank, accession number OR635616. From the sample that tested DCH positive on cPCR only, no additional sequence could be obtained.

### 3.3. Phylogenetic Analysis of DCH Chile Sequence

**Figure 1:**
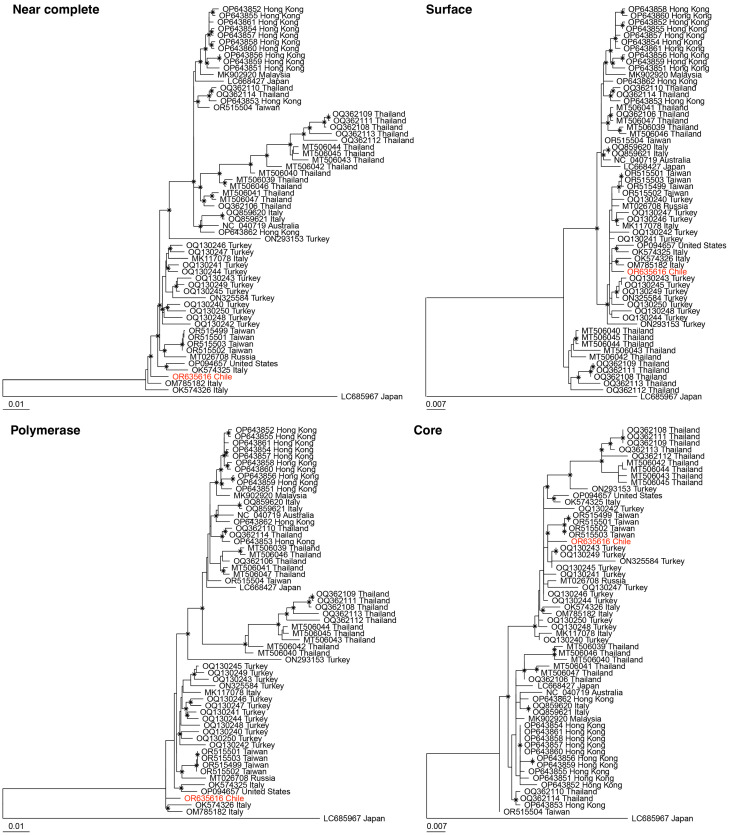
Maximum likelihood phylogenetic analysis of nucleotide alignments of the near-complete genome (3197 nt), polymerase (2526 nt), surface (1152 nt) and core (657 nt) proteins of 59 unique domestic cat hepadnaviruses downloaded from NCBI GenBank. The domestic cat hepadnavirus sequence detected in this study is presented in red. The virus sequences are named by NCBI GenBank accession number followed by the country in which the virus was detected. Bootstrap support >70% is represented by the asterisk symbol on the branch node, and the tree is rooted at midpoint for clarity. The scale bar represents the number of nucleotide substitutions per site.

The phylogenetic positioning of the DCH detected in this study varied depending on whether phylogenetic analysis was based on the whole near-complete genome, polymerase, surface or core protein sequences (Figure 1). In the near-complete genome and polymerase protein phylogenies, DCH_Chile branches off from the main cluster of DCH strains, with 71% bootstrap and 37% bootstrap support for the near-complete and polymerase phylogenies, respectively. For the surface protein trees, DCH_Chile forms a clade with three DCH strains detected in Italy, and one detected in the United States within the larger clustering of DCH strains (bootstrap support for this position is only 47%). In contrast, DCH_Chile clusters with three DCH strains from Taiwan in the core protein tree with adequate bootstrap support (73%).

This variability in clustering raised the possibility of DCH recombination, a phenomenon that has been described in human HBV. We analysed this using recombination detection program software (RPD5 version Beta 5.53) employing RDP, GENECONV, Bootscan, SiScan, 3Seq and Chimaera analysis [29,30,31,32,33,34]. The RDP5 analysis showed no recombination event within the DCH_Chile near-complete genome.

### 3.4. Retrovirus Infections in Chilean Cats

**Figure 2:**
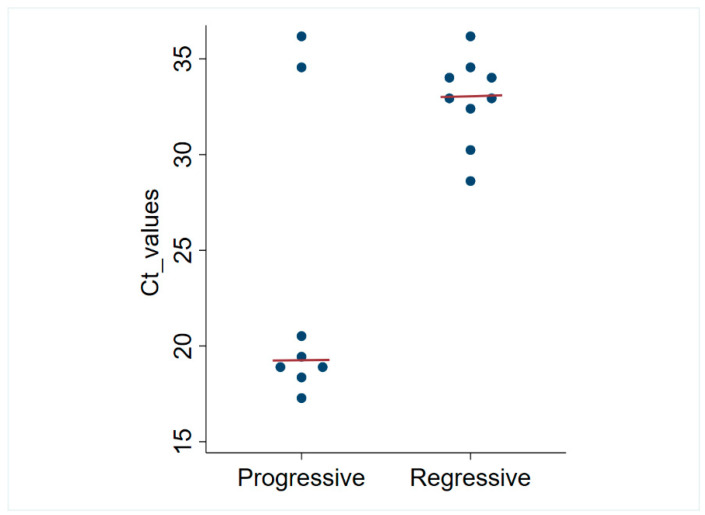
Dot plot of FeLV qPCR Ct values for progressive and regressively infected cats in Santiago, Chile. The horizontal lines represent the median values.

The results of retrovirus testing are shown in Table 3. In total, 8/120 (6.7%) progressive (antigen-positive, provirus-positive) FeLV infections, including 2 coinfections with FIV, were detected, and 9/120 (7.5%) regressive (antigen-negative, provirus-positive) FeLV infections were detected. The distribution of Ct values for progressive and regressively infected cats is depicted in Figure 2. The median Ct value was 19.33 (range: 17.44 to 35.96) for progressive infections, which was significantly lower than 33.05 (range: 28.66 to 35.98) for regressive infections (*p* = 0.048).

FeLV nPCR was carried out on 8/8 samples with progressive FeLV infection, and on 3 samples with regressive infection that had the highest Ct values. FeLV nPCR was positive in all samples, and the identity of the product was confirmed with Sanger sequencing, where the top 100 hits were FeLV, with query coverage and nucleotide identity greater than 90%, E value 0. The only independent variable significantly associated with progressive FeLV infection was male sex, as presented in Table 4. Robust statistical comparison was not possible for breed and neuter status due to highly imbalanced group sizes.

For FIV, 3/120 cats tested FIV positive on serological testing (2.5%), of which two were dual infections with FeLV (Table 3). No DCH and retrovirus co-infections were identified.

## 4. Discussion

This study demonstrates that DCH is circulating among cats in Chile, thereby expanding the list of regions reporting cats infected with this novel hepadnavirus. The frequency of molecular detection of DCH in this study (2/120, 1.67%) is similar to that reported in the USA (1/496, 0.2%) [10], Japan (2/203, 0.99%) [35], and the United Kingdom (2/108, 1.9%) [11]. In contrast, higher molecular prevalences are reported in Australasia, including from Australia (8/123, 6.5%; 7/67, 10.4%), Italy (42/390, 10.8%), Thailand (26/209, 12.4%), Malaysia (31/253, 12.3%) and Hong Kong (57/513, 11.1%) [4,5,6,7,8,10].

The total number of DCH-infected cats is expected to be greater than indicated by detection of viral DNA. In humans, circulating HBV DNA is a proxy for viraemia and is detected in both acute and chronic infections [36]. However, many HBV-infected humans will suppress viraemia, either through immune control (inactive HBeAg-negative phase) or functionally clearing the infection (measured by HBsAg loss). In either case, individuals do not completely eliminate covalently closed circular DNA (cccDNA) from the liver but remain persistently HBV infected. Hence, markers other than circulating DNA are required to identify all infected patients. Antibodies recognizing viral core proteins (anti-HBc) are a useful marker of HBV exposure and persistent infection, appearing early after HBV infection (IgM) and persisting (IgG), usually for the patient’s lifetime [37]. Similarly, in cats, Fruci et al. detected DCHcAbs in 64/256 (25%) sera, when only 25/256 (9.8%) were positive by qPCR [38], supporting that molecular surveys underestimate the true prevalence of DCH infection. Even though we did not detect all DCH infections, a pattern may be emerging of regions with high (Australia, Asia and Southern Europe) and low (North and South America, Japan and UK) prevalence, where Chile would group with the latter.

BLAST nt sequence comparison of the near-complete genome and partial polymerase protein (49 bp missing from the 3′ end) of DCH_Chile (accession number: OR635616) detected in this study showed 98.9% sequence identity to the closest related DCH strain cat41-19/Italy (accession number OM785182.2). Sequence comparison of the surface protein and partial core protein (132bp missing from the 5′ end) showed identities of 99.4% and 99% to the closest related DCH strains with complete sequences TR-744 (accession number: OQ130250.1) and TR-409 (accession number: OQ130250.1), respectively. Phylogenetic analysis based on nucleotide alignments showed that the DCH_Chile sequence occupied contrasting positions within the phylogeny depending on whether the near-complete genome or individual proteins were analysed. No recombination event was detected using RDP5. However, the DCH_Chile sequence is missing a 361bp section of the genome that makes up part of the polymerase and C protein. It is possible that this missing genome section contains a recombination breakpoint.

Our study used concurrent FeLV antigen and qPCR testing for FeLV provirus, which allows the frequency of progressive and regressive infections to be estimated. Our results for FeLV testing can be compared to those from a recent cross-sectional study from Europe [14]. In that study, Italy was identified as having a very high FeLV prevalence, 21/269 (7.8%) progressive, 12/269 (4.5%) regressive, and Germany as having a low prevalence with 6/318 (1.9%) progressive and 4/318 (1.3%) regressive infections. Our findings (8/120 progressive (6.6%) and 9/120 (7.5%) regressive) are similar to those from Italy and suggest that progressive FeLV infection is a significant cause of disease among cats in urban Chile. Male cats were at greater risk of progressive FeLV infection than females (8/54 male cats, 0/66 females). This result should be interpreted cautiously as there was a zero cell. Nonetheless, a male predisposition for progressive FeLV infection is consistent with the findings of others, including a recent study from Brazil, which is the only other study in South America to investigate progressive FeLV infection [16], and others [39]. Of the 8/120 progressively infected cats (6.6%), most or all are expected to develop FeLV-related diseases. Further, two of these eight individuals (1.6%) were concurrently FIV-infected, a combination that carries a much worse prognosis than even progressive FeLV infection [19]. One other FIV infection was identified here, giving an overall frequency of FIV infection of 2.5%.

Free-roaming domestic cats in remote areas of Chile have been prioritized in previous studies to investigate potential feline pathogens shared between domestic cats and sympatric guignas (Leopardus guigna), Chile’s threatened, small leopard cat. These studies used either serology or PCR, but not both, for retrovirus detection in domestic cats. In a serological study of owned cats in remote Valdivia, southern Chile 17/124 (13.7%) tested FeLV antigen positive, 14/124 (11.3%) had FIV antibodies, and 3/124 (2.4%) tested dual FeLV/FIV positive [40]. A similar study targeting owned, free-roaming cats in Valdivia and Chiloe Island reported that 10/112 (8.9%) and 2/112 (1.7%) tested seropositive for FeLV or FIV, respectively [41]. Using nPCR for FeLV and FIV, respectively, 26/78 (33.3%) and 2/78 (2.5%) domestic cats from Chiloe Island tested positive [42]. Finally, a study from Chile’s Mediterranean bioclimatic area, which includes Santiago, found 9/44 (20.5%) domestic cats tested positive for FeLV by nPCR [43]. In our study, the total number of FeLV qPCR positive samples was 17/120 (14.2%), which, although the studies cannot be directly compared and sample sizes are small, suggests that the burden of FeLV may be higher in free-roaming cats from remote areas of Chile than from the Greater Santiago area. On the other hand, the frequency of FIV seropositive cats is similar between our study and previous studies in Chile and is low in comparison to most other regions [18].

Limitations of our study include the small sample size, which precluded the analysis of some risk factors, especially for DCH detection and FIV infection. Outcomes for FeLV infection could be more completely defined by using a longitudinal rather than cross-sectional study, and by including additional testing modalities, such as antibody testing.

## 5. Conclusions

Progressive FeLV infection is common in domestic cats in urban Chile; testing and control measures for this pathogenic retrovirus are indicated. In addition, the global footprint of DCH includes Chile. The potential impact of FeLV, FIV and DCH on Chile’s wildcat species is worthy of further investigation.

## Figures and Tables

**Table 3:** Results of infectious disease testing for 120 domestic cats from Chile.

Combined Testing Results					
FeLV p27 Antigen	FeLV qPCR	FIV Antibody	DCH cPCR	Frequency (%)	Interpretation of Test Results
Positive	Positive	Negative	Negative	6/120 (5%)	Progressive FeLV infection
Positive	Positive	Positive	Negative	2/120 (1.7%)	Dual FeLV/FIV infected
Negative	Positive	Negative	Negative	9/120 (7.5%)	Regressive FeLV infection
Positive	Negative	Negative	Negative	1/120 (0.8%)	False positive FeLV antigen result
Negative	Negative	Positive	Negative	2/120 (1.7%)	FIV infected
Negative	Negative	Negative	Positive	2 */120 (1.7%)	DCH infected
Negative	Negative	Negative	Negative	98/120 (81.7%)	None of the infectious agents detected

* One of two DCH cPCR positive samples also tested DCH qPCR positive.

**Table 4:** Results of FeLV qPCR and antigen testing their association with the independent variables of interest amongst the studied cats in Chile.

Variable	Categories	Progressive FeLV Infection(qPCR and p27 Positive) (%)	*p* Value
Age	≤3 years old	2/26 (7.69%)	1.000
	>3 years old	6/82 (7.32%)	
Sex	Female	0/66 (0.00%)	0.001 *
	Male	8/54 (14.81%)	
Environment	access to outdoors	7/94 (7.45%)	1.000
	Indoors only	1/26 (3.85%)	
Source	Client-owned	8/95 (8.42%)	0.202
	Shelter-owned	0/25 (0.00%)	
Health status	Healthy	5/83 (6.02%)	0.699
	Sick	3/37 (8.11%)	

* Significant association found at *p* = 0.05.

## Data Availability

Data are contained within the article.
